# *Leishmania tarentolae*: A new frontier in the epidemiology and control of the leishmaniases

**DOI:** 10.1111/tbed.14660

**Published:** 2022-08-03

**Authors:** Jairo Alfonso Mendoza‐Roldan, Jan Votýpka, Claudio Bandi, Sara Epis, David Modrý, Lucie Tichá, Petr Volf, Domenico Otranto

**Affiliations:** ^1^ Department of Veterinary Medicine University of Bari Valenzano Italy; ^2^ Department of Parasitology, Faculty of Science Charles University Prague Czech Republic; ^3^ Biology Centre, Institute of Parasitology Czech Academy of Sciences České Budějovice Czech Republic; ^4^ Department of Biosciences and Pediatric CRC “Romeo ed Enrica Invernizzi” University of Milan Milan Italy; ^5^ Department of Botany and Zoology, Faculty of Science Masaryk University Brno Czech Republic; ^6^ Department of Veterinary Sciences, Faculty of Agrobiology, Food and Natural Resources Czech University of Life Sciences Prague Czech Republic; ^7^ Department of Pathobiology, Faculty of Veterinary Science Bu‐Ali Sina University Hamedan Iran

**Keywords:** leishmaniasis/leishmaniases, *Leishmania infantum*, *Leishmania tarentolae*, *Sauroleishmania*, *Sergentomyia*, vaccine

## Abstract

Leishmaniasis (or the leishmaniases), classified as a neglected tropical parasitic disease, is found in parts of the tropics, subtropics and southern Europe. *Leishmania* parasites are transmitted by the bite of phlebotomine sand flies and million cases of human infection occur annually. *Leishmania tarentolae* has been historically considered a non‐pathogenic protozoan of reptiles, which has been studied mainly for its potential biotechnological applications. However, some strains of *L. tarentolae* appear to be transiently infective to mammals. In areas where leishmaniasis is endemic, recent molecular diagnostics and serological positivity to *L. tarentolae* in humans and dogs have spurred interest in the interactions between these mammalian hosts, reptiles and *Leishmania infantum*, the main aetiologic agent of human and canine leishmaniasis. In this review, we discuss the systematics and biology of *L. tarentolae* in the insect vectors and the vertebrate hosts and address questions about evolution of reptilian leishmaniae. Furthermore, we discuss the possible usefulness of *L. tarentolae* for new vaccination strategies.

## 1 THE STORY OF TWO SISTERS: REPTILIAN AND MAMMALIAN LEISHMANIAS

Trypanosomatids of the genus *Leishmania* (Kinetoplastida, Trypanosomatidae) are responsible for a significant health burden to mammals, including humans, in many tropical, subtropical and temperate regions, with 20 *Leishmania* spp. associated with human diseases (Okwor & Uzonna, 2016; Otranto & Dantas‐Torres, 2013). For example, zoonotic visceral leishmaniasis caused by *Leishmania infantum* is a neglected disease of medical and veterinary importance worldwide, with the agent being transmitted by sand flies of the genera *Phlebotomus* in the Old World (Maroli et al., 2013) and *Lutzomyia* in the New World (Dantas‐Torres et al., 2012).

A group of 21 less‐studied leishmaniae, belonging to *Sauroleishmania*, is usually associated with sand flies of the genus *Sergentomyia*, which have long been considered to feed primarily on cold‐blooded vertebrates (Akhoundi et al., 2016). Among them, *Leishmania* (subgenus *Sauroleishmania*) *tarentolae* was described from the gecko *Tarentola mauritanica* in Europe, North Africa and the Middle East (Telford, 2009). Although it has long been considered non‐pathogenic and specific to its reptilian hosts, some strains of *L. tarentolae* (e.g., the strain LEM‐125) were shown under laboratory conditions to cause transient infections in mammalian cells, differentiating into the amastigote stage, but not efficiently replicating within mammalian macrophages (Adler, 1962; Breton et al., 2005; Novo et al., 2015; Taylor et al., 2010). However, the unexpected detection of *L. tarentolae* in a mummy (Novo et al., 2015) and in human blood (Iatta et al., 2021; Pombi et al., 2020) triggered further investigations of the role of this trypanosomatid in the context of the leishmaniases and their control. Other members of the subgenus *Sauroleishmania*, such as *Leishmania adleri*, have also been associated with cutaneous leishmaniasis in humans (Coughlan et al., 2017; Manson‐Bahr & Heisch, 1961), reflecting the understudied status of *Sauroleishmania*. Moreover, understanding the biology of *L. tarentolae* is highly relevant, given the myriad of applications in biotechnology due to (i) apparent absence of pathogenicity for humans and other mammals, (ii) easy and inexpensive cultivation and (iii) robustness as a platform for the production of recombinant proteins (Klatt et al., 2019; Niimi, 2012). For example, *L. tarentolae* exhibits mammalian‐like post‐translational modifications, which makes it a useful source for expressing functional mammalian antibody fragments and human glycoproteins (Jørgensen et al., 2014; Klatt & Konthur, 2012), such as N‐glycans erythropoietin (Cantacessi et al., 2015) and amyloid precursor protein alpha (Klatt et al., 2013). Importantly, the finding of *L. tarentolae* in dogs, reptiles (i.e., both geckos and lizards), sand flies and humans in the same area where *L. infantum* is endemic (2021aIatta et al., 2021; Mendoza‐Roldan et al., 2021, 2022; Pombi et al., 2020) opens many questions about the interactions between both trypanosomatid flagellates, potentially offering new opportunities for vaccines and/or immune‐protection strategies to control canine and human leishmaniases. This review provides a comprehensive account of the main features of *L. tarentolae* systematics, phylogenetics and evolution, along with its biology in the insect vectors and the vertebrate hosts.

## 2 ORIGIN, EVOLUTION AND SYSTEMATICS OF SAURIAN‐ASSOCIATED *LEISHMANIA*

The genus *Trypanosoma* has long been considered the most basal trypanosomatid branch, supporting the dixenous origin of this family. However, the branching of the recently described monoxenous flagellate *Paratrypanosoma confusum* between free‐living bodonids and parasitic trypanosomatids (Flegontov et al., 2013), favours the insect‐first scenario, in which the ancestral flagellate first invaded insects, and then only subsequently colonized vertebrate hosts, probably through blood feeding (Lukeš et al., 2018). The derived dixenous lifestyle evolved from the monoxenous one several times independently, initially in *Trypanosoma* and later on in the two‐host genera *Leishmania* and *Phytomonas*, which are phylogenetically nested within the monoxenous trypanosomatids (Lukeš et al., 2014; Lukeš et al., 2018, 2014; Maslov et al., 2013).

**FIGURE 1:**
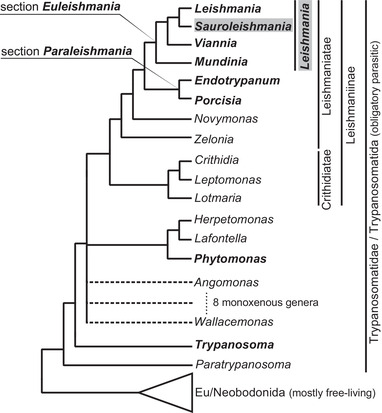
A schematized tree summarizing multiple phylogenetic reconstructions, mostly 18S rRNA gene‐based and showing relationships been monoxenous and heteroxenous (in bold) trypanosomatids and between *Leishmania* four subgenera

Despite the fact that *Leishmania* spp. have been intensively studied, there are many open questions regarding their taxonomy and phylogeny. Both concepts recently underwent substantial changes described below (Cupolillo et al., 2000; Espinosa et al., 2018; Harkins et al., 2016; Klatt et al., 2019; Kostygov & Yurchenko, 2017; Kostygov, et al., 2021). All *Leishmania* spp. belong to the subfamily Leishmaniinae within the family Trypanosomatidae in the order Trypanosomatida (Figure 1). On closer examination, leishmaniae are grouped together with the newly described monoxenous genera *Novymonas*, *Borovskyia* and *Zelonia* in the infrafamily Leishmaniatae, while two established and species‐rich monoxenous genera, *Leptomonas* and *Crithidia*, together with *Lotmaria* form the infrafamily Crithidiatae (Figure 1). With monophyly well supported, all dixenous leishmaniae form two major sister lineages informal designated as sections or divisions: section *Paraleishmania* brings together the genera *Endotrypanum* and *Porcisia* (formerly *Paraleishmania*; see Kostygov & Yurchenko, 2017), while the genus *Leishmania* belongs to the section *Euleishmania* (the true *Leishmania*). Members of this genus are further divided into four subgenera: *Leishmania*, *Viannia*, *Sauroleishmania* and *Mundinia* (formerly the *Leishmania enriettii* complex) (Figure 1). The subgenus *Sauroleishmania* was established half a century ago, although its type species *L. tarentolae* was described much earlier (Wenyon, 1920), and includes more than 20 species, which are restricted to the Old World (Akhoundi et al., 2016). *Sauroleishmania* spp. are known as reptilian parasites that have been consistently detected in various reptiles belonging to the saurian families Agamidae, Gekkonidae, Lacertidae, Scincidae and Varanidae originating from Mediterranean Europe, North Africa and the Middle East (Telford, 2009; Wilson and Southgate, 1979), yet there are some interesting exceptions. Unlike most *Sauroleishmania* spp., *L. adleri* is capable of infecting mammals (Coughlan et al., 2017) and causes transient skin symptoms in humans (Manson‐Bahr & Heisch, 1961) and asymptomatic infections in hamsters and mice (Adler, 1962). An undescribed species of *Sauroleishmania* (different from *L. adleri* and *L. tarentolae*) was found to cause visceral leishmaniasis in humans and dogs in China (Chen et al., 2019; Yang et al., 2013). Moreover, *L. tarentolae* promastigotes are capable of invading mammalian (including human) dendritic cells (DC) and macrophages, where they differentiate into an amastigote‐like form, yet there is no unambiguous evidence of their replication (Breton et al., 2007; Taylor et al., 2010).

There are three mutually exclusive hypotheses postulating the origins of the genus *Leishmania* from the Palearctic or the Neotropics, or from the supercontinent before its split into present continents (Akhoundi et al., 2016; Harkins et al., 2016; Klatt et al., 2019; Lukeš et al., 2007; Schönian et al., 2018). The oldest fossil record of a protist parasite is represented by *Paleoleishmania proterus* found in the midgut lumen of a blood‐filled female of the sand fly *Palaemyia burmitis* entrapped in mid‐Cretaceous amber (∼100 MYA) in Myanmar (Poinar, 2004; Poinar & Poinar, 2004a). Promastigotes were mixed with nucleated reptilian blood cells, likely representing the ancestor of the genus *Sauroleishmania* (Poinar & Poinar, 2004a, 2004b). This finding implies that *Sauroleishmania* forms a sister clade to all other *Leishmania* species. However, the phylogenetic position of *Sauroleishmania* between the mammal‐infecting subgenera *Leishmania* and *Viannia* suggests that this species‐rich subgenus switched from mammals to reptiles (Klatt et al., 2019; Schönian et al., 2018). Although the available fossil record supports reptiles as early hosts of *Leishmania*‐like parasites, the reptile‐infecting subgenus *Sauroleishmania* must have arisen later, after the adaptation of *Leishmania* to mammals. While subsets of data can be used to support each of these hypotheses, the prevailing view places the origin of *Leishmania* in the Mesozoic, prior to the breakup of Gondwana.

## 3 GUT FEELING: *LEISHMANIA TARENTOLAE* DEVELOPMENT IN A SAND FLY GUT

*Sauroleishmania* spp. are generally transmitted by reptile‐biting sand flies of the genus *Sergentomyia*, with many species found infected by various *Sauroleishmania* species (Karimi et al., 2014; Killick‐Kendrick et al., 1986; Maroli et al., 1988; Rashti & Mohebali, 1994). Although *Sergentomyia* spp. feed primarily on reptiles, some species have been reported to bite mammals, including humans, raising a question about the role of these vectors in the transmission of mammal‐infecting *Leishmania* species, particularly *L. infantum* (Maia & Depaquit, 2016). Nevertheless, involvement of other sand fly genera in *Sauroleishmania* transmission should also be considered. Indeed, *L. tarentolae* DNA was recently detected in *Phlebotomus perfiliewi* (Pombi et al., 2020), *Phlebotomus perniciosus* (Latrofa et al., 2021; Mendoza‐Roldan et al., 2021) and heavy late‐stage infections were demonstrated experimentally in *Phlebotomus papatasi* (Adler & Theodor, 1929), *P. perniciosus* and *Phlebotomus sergenti* (Ticha et al., 2021). This may be due to the fact that many *Phlebotomus* species are opportunistic feeders, and their host‐seeking behaviour may vary depending on the location, season and host availability (Quate, 1964). Their willingness to feed on cold‐blooded animals has been repeatedly documented, with a prominent case of *P. papatasi* (Adler & Theodor, 1929; Belova, 1971; Quate, 1964), which is susceptible to *Sauroleishmania* spp. infection (Adler & Theodor, 1929; Ticha et al., 2021). Collectively, these data suggest that sand flies of the genus *Phlebotomus* may play a role as alternative vectors in the circulation of *L. tarentolae*, and therefore in its transmission to non‐reptilian hosts (Ticha et al., 2021). As *Sergentomyia* is a genus exclusively present in the Old World, the transmission cycle of *L. tarentolae* in Brazil must be due to other vectors, possibly by *Lutzomyia* spp., as *L. tarentolae* was shown to develop in *Lutzomyia longipalpis* under laboratory conditions (Diaz‐Albiter et al., 2018).

**FIGURE 2:**
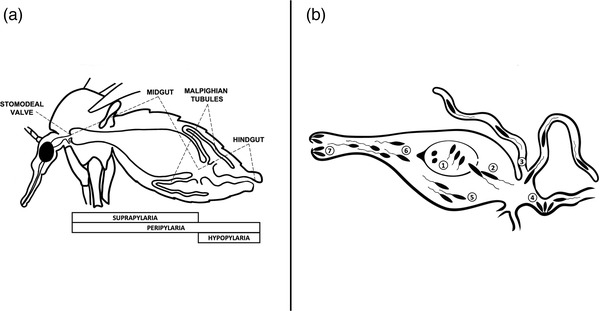
Sand fly digestive tract and summary Suprapylaria/Peripylaria/Hypopylaria (a) and development of *Leishmania tarentolae* (b)

Based on their development in vectors, Lainson and Shaw (1987) classified parasites of the genus *Leishmania* into three groups (Figure 2(a)). The Suprapylaria (subgenus *Leishmania*) includes mammal‐infecting species of the Old World (e.g., *L. infantum*), whose development is restricted to the midgut. The Peripylaria (subgenus *Viannia*) encompasses mammal‐infecting species of the New World (e.g., *Leishmania braziliensis*), which develop in the hindgut and then migrate to anterior midgut. The third group consisting of reptile‐infecting species (subgenus *Sauroleishmania*, including *L. tarentolae*) was named as Hypopylaria (Figure 2(a)). The development of these parasites was believed to be limited to the hindgut, suggesting that transmission to reptiles occurs when infected sand fly is ingested. In contrast, species with suprapylarian or peripylarian type of development are transmitted to mammals by sand fly bites (Bates, 2007).

However, some *Sauroleishmania* species are capable of an anterior migration in the sand fly gut, associated with colonization of the anterior midgut (Figure 2(b)) (Adler & Theodor, 1929, 1935; Ticha et al., 2021) and it is assumed that the hypopylarian type of development occurs only in some *Sauroleishmania*‐sand fly combinations. The development seems to be influenced by the insect, since *L. tarentolae* undergoes the peripylarian type of development in *P. papatasi* and *P. perniciosus*, yet the hypopylarian development prevails in *P. sergenti* (Ticha et al., 2021). Such variability in the vector‐parasite interaction may be due to different behaviour of *Sauroleishmania* species to escape from the blood meal surrounded by the peritrophic matrix and by their capacity to attach to different parts of the sand fly gut.

It has also been proposed that the hypopylarian type of development occurs when promastigotes cannot cross the peritrophic matrix and are passed into the hindgut (Bates, 2007). Indeed, the role of peritrophic matrix in parasite life cycle is important. For example, its delayed degradation in *Sergentomyia schwetzi* is known to cause the refractoriness of this vector to mammal‐infecting *Leishmania* species (Sádlová et al., 2018). However, further studies on *Sauroleishmania*‐sand fly interactions are necessary to confirm these hypotheses.

Although *S. minuta*, the proven natural vector of *L. tarentolae*, is one of the most abundant sand flies in the Mediterranean (Maroli et al., 1988), only two studies described the development of *L. tarentolae* in this sand fly species (Adler & Theodor, 1935; Telford, 2009). Females of *S. minuta* (erroneously referred to as *Phlebotomus parroti* in the original description (Telford, 2009) were experimentally infected by feeding on gecko *T. mauritanica* carrying a mixed infection of *L. tarentolae* and *Trypanosoma platydactyli*. Both parasites acquired an anterior position in the sand fly gut, with *Sauroleishmania* promastigotes found in the midgut and cardia, but not in the hindgut (Adler & Theodor, 1935). Recently, the development of *L. tarentolae* in Malpighian tubules of three *Phlebotomus* species was experimentally demonstrated (Ticha et al., 2021). The localization in Malpighian tubules is rather unique for the genus *Leishmania*, with only two other reports of unidentified promastigotes in *Sergentomyia garnhami*, *Sergentomyia antennata* (Kaddu, 1986) and in *S. minuta* (Killick‐Kendrick et al., 1979). An examination of laboratory bred *S. minuta* females that were allowed to feed on naturally infected geckos revealed that *L. tarentolae* is able to colonize the Malpighian tubules of both *Sergentomyia* spp. and *Phlebotomus* spp. (Ticha et al., unpublished). Though there are only few records of *Sauroleishmania* morphological forms in vectors (Adler & Theodor, 1929, 1935), they do not differ from those known for *Leishmania* in mammals, but the infectious stages for reptiles are not known (Bates, 2007). So far, a successful experimental transmission of *Sauroleishmania* from sand flies to reptilian hosts has not been demonstrated.

**FIGURE 3:**
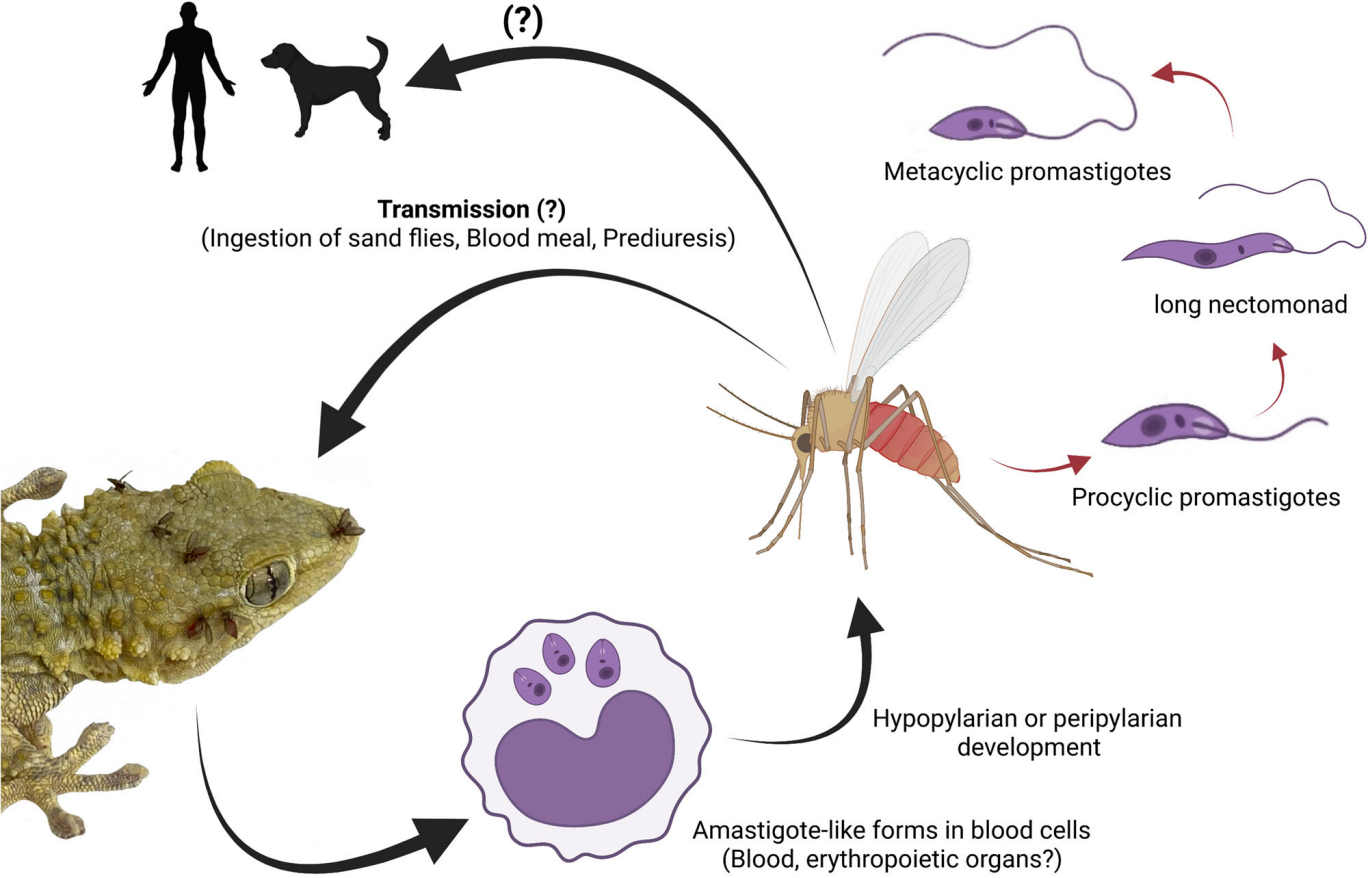
Life cycle of *Leishmania tarentolae* in vectors and hosts. In reptiles, amastigote‐like forms develop in blood cells, and parasite DNA has been detected in blood and erythropoietic organs. Sand flies ingest infected blood cells and parasites differentiate into promastigote forms and undergo hypopylarian or peripylarian type of development. Possible transmission routes to vertebrate hosts are via sand fly bite, oral ingestion of the fly or contaminative way by prediuresis. Transmission and development in mammals are not known

Two possible modes of transmission may be considered. The hypopylarian type of development of some *Sauroleishmania* species suggests that reptiles become infected by ingestion of a sand fly. On the contrary, species with the peripylarian type might be transmitted by bite, via the pool‐blood feeding mechanism, similarly to mammal‐infecting *Leishmania* (Bates, 2007). Colonization of the stomodeal valve and disruption of its surface are essential for effective transmission of *Leishmania* to its mammalian hosts, as it facilitates the regurgitation of parasites from the midgut (Dostálová & Volf, 2012). The presence of *L. tarentolae* promastigotes in the cardia and colonization of the stomodeal valve in *Phlebotomus* spp. (Adler & Theodor, 1929; 1935) support the idea of transmission by bite (Figure 3). However, the localization of *L. tarentolae* in Malpighian tubules raises a third possible scenario, namely the transmission by prediuresis. When feeding on a host, sand fly females regularly excrete urine to concentrate proteins in bloodmeal and restore weight and water balance (Sádlová et al., 2013). Viable *L. major* promastigotes, including the metacyclic form, were found in urine droplets discharged by infected *P. papatasi* and *Phlebotomus duboscqi* females, during feeding (Sádlová & Volf, 1999). *Leishmania* promastigotes in urine droplets may enter bite wounds or mucosal membranes. As urine is secreted from Malpighian tubules and passes the hindgut (both tissues being the typical location of *L. tarentolae* promastigotes), the role of prediuresis in *Sauroleishmania* transmission should be considered plausible and therefore further studied.

## 4 MAMMALIAN EXPOSURE TO *LEISHMANIA TARENTOLAE* AND THE ROLE OF REPTILES IN THE LEISHMANIASES

In the early years, *L. tarentolae* was classified as *Leptomonas* while researchers investigated *T. mauritanica* gecko as a possible reservoir of a zoonotic disease called Biskra boil and caused by *Leishmania* spp. (Sergent et al., 1914). Soon after, while describing different types of reptilian flagellates from Egypt, Wenyon (Wenyon, 1920) mentioned that the species isolated by Sergent (Sergent et al., 1914) was in fact a *Leishmania*, later becoming *L. tarentolae*. At the moment of the first isolation of *L. tarentolae*, some authors hypothesized that geckos could be reservoirs of cutaneous leishmaniasis caused by *Leishmania tropica* and/or *Leishmania major* (Chatton & Blanc, 1914; McMillan, 1966; Sergent et al., 1914; Wenyon, 1920). Also, other *Sauroleishmania* species were suspected to be causative agents of cutaneous leishmaniasis or oriental sore. For example, *L. adleri* was isolated from the blood of *Latastia longicaudata* lizards in Kenya (Heisch, 1958), and was believed to be a strain of *Leishmania donovani*. Unlike *L. tarentolae*, more studies confirmed the pathogenic effect of *L. adleri* as the causative agent of cutaneous leishmaniasis in rodents and humans (Coughlan et al., 2017; Manson‐Bahr & Heisch, 1961). It was hypothesized that interactions between mammalian and reptilian leishmania (i.e., *L. tarentolae* in mammals and *L. donovani* in reptiles) could ultimately result in partial dilution of species, thus immunization and protection, within the two sister clades (Mutinga & Ngoka, 1981).

**FIGURE 4:**
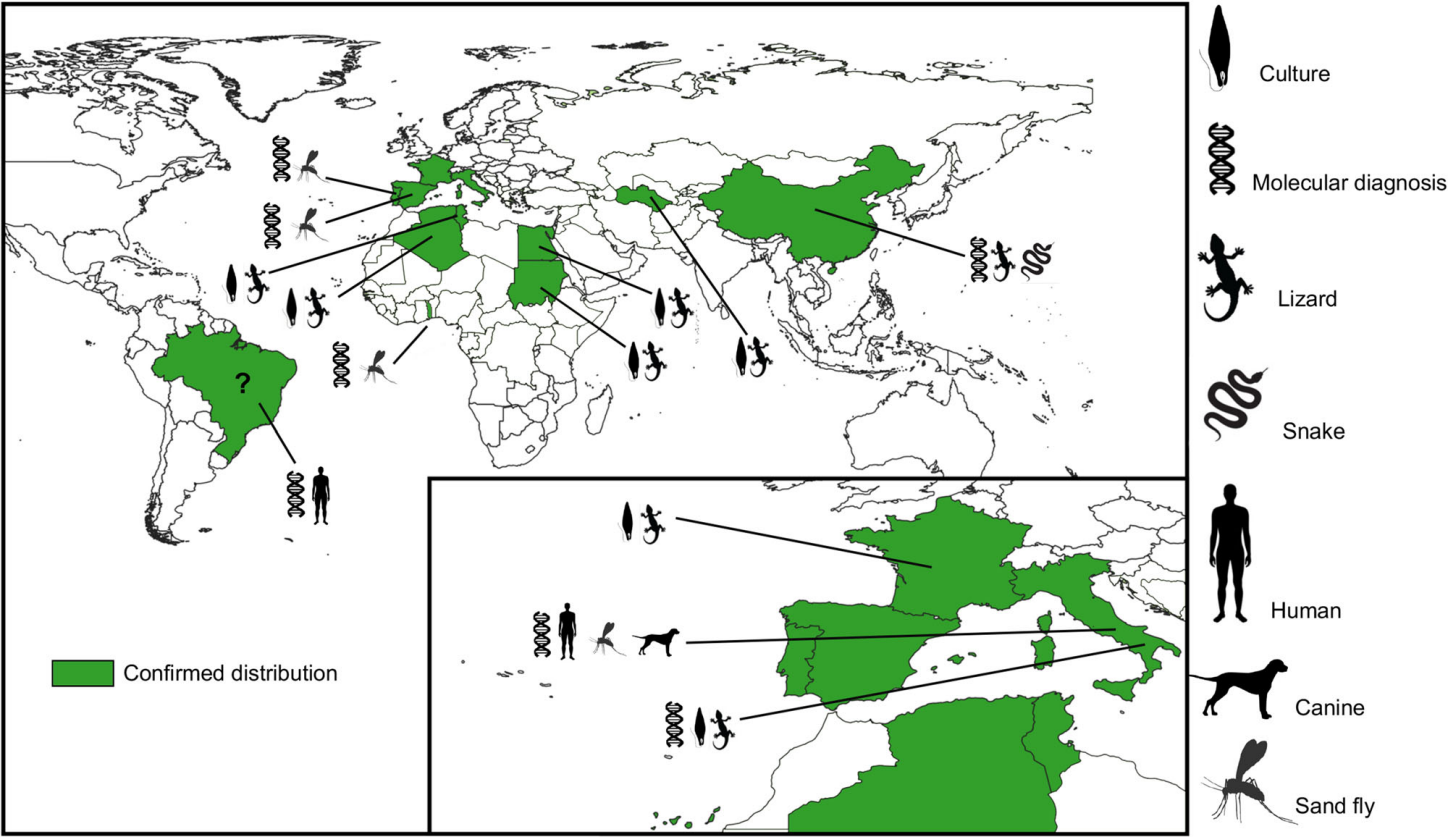
Distribution map of *Leishmania tarentolae* based on isolates and molecular detection in reptiles, sand flies and mammals (Annex 1). Dark green represents confirmed distribution; question mark refers a controversial finding concerns parasites detected in bone marrow and intestinal tissue samples from a 300‐year‐old Brazilian mummy based on a kDNA amplicon matching to *L. tarentolae* (Novo et al. 2015), which, however, does not agree with the geographical distribution of the subgenus *Sauroleishmania*

Furthermore, additional attempts were made to identify and isolate *Sauroleishmania* from endemic areas of human and canine leishmaniasis. Axenic cultures of *L. tarentolae* were obtained from France (Gao et al., 2001) and Italy (Mendoza‐Roldan et al., 2022; Pozio, et al., 1983) with reports of *L. tarentolae* in different species of reptiles (Klatt et al., 2013; Klatt et al., 2019; Mendoza‐Roldan et al., 2022), sand flies (Mendoza‐Roldan et al., 2021) and mammals (Iatta et al., 2021) (Figure 4; Novo et al., 2015; Pombi et al., 2020; Annex 1). In particular, *L. tarentolae* is widely distributed and can infect saurian reptiles from the Gekkonidae (i.e., *Mediodactylus kotschyi*, *Tarentola annularis*, *T. mauritanica*) and the Lacertidae (i.e., *Podarcis filfolensis*, *Podarcis siculus*) families in the Mediterranean context (Figure 4; Annex 1) (Elwasila, 1988; Klatt et al., 2013; Mendoza‐Roldan et al., 2022; Pozio et al., 1983).

While studying the molecular prevalence of *L. infantum* in human donors, sand flies and dogs from central Italy, *L. tarentolae* was detected by nested‐PCR in humans and sand flies (i.e., *Phlebotomus* and *Sergentomyia*) (Pombi et al., 2020). This finding was most likely related to the *Sergentomyia* spp. transmitting *L. tarentolae* while feeding on humans (Mendoza‐Roldan et al., 2021; Pombi et al., 2020). Moreover, the substantial reduction in anti‐*L. infantum* antibody titres of more than half of the population of *L. infantum*‐seropositive and clinically healthy sheltered dogs, sampled throughout the year (that is, during the transmission and non‐transmission season), raised questions about the possibility of dogs being exposed to *L. tarentolae* (Cavalera et al., 2021). In fact, circumstantial evidence suggested by the seasonal variation in antibody levels depending on the sand fly activity and sympatric occurrence of *L. tarentolae* and *L. infantum* could possibly indicate a protective effect of the exposure to *L. tarentolae* in areas endemic to canine leishmaniasis, reducing the clinical manifestation of leishmaniasis in dogs. The likelihood of infection by *L. tarentolae* in mammals was further confirmed serologically and molecularly in southern Italy, both in humans (Iatta et al., 2021) and in sheltered dogs (Mendoza‐Roldan et al., 2021). Moreover, the finding of *S. minuta* as the most abundant species in canine leishmaniasis endemic areas (Mendoza‐Roldan et al., 2021; Pombi et al., 2020), further suggested the possibility of mammalian exposure to *L. tarentolae*, also considering the feeding behaviour of this sand fly species on humans and dogs.

Capability of pathogenic mammalian‐associated *Leishmania* to infect reptiles was studied in the late 1960s and 1970s and was ultimately disregarded, mainly given the physiological differences between mammals and reptiles (e.g., reptiles being ectotherms and mammals endotherms) (Belova, 1971; McMillan, 1966). Nevertheless, Belova (1971) described experimental infections of reptiles with mammalian‐associated *Leishmania* spp., and this was later confirmed by molecular detection of various *Leishmania* spp. (i.e., *L. donovani*, *L. tropica*, *L. turanica*) in saurians and snakes in China (Chen et al., 2019; Zhang et al., 2019). Furthermore, *L. infantum* was molecularly detected in lizards in areas of canine leishmaniasis in southern Italy, in sympatric occurrence with *L. tarentolae* (Mendoza‐Roldan et al., 2022). The infection of *L. infantum* in reptiles was further corroborated through the retrieval of amastigote forms in the bone marrow of geckoes (Mendoza‐Roldan et al., 2022). These molecular findings suggest the interaction between both *Leishmania* species and ultimately raise the question who was infected first – reptiles by *Leishmania* or mammals by *Sauroleishmania*?

## 5 *LEISHMANIA TARENTOLAE* AND THE CELLULAR MODEL

*Leishmania tarentolae* is broadly used for a range of biotechnological applications, from protein production to its exploitation as a model for drug discovery (Klatt et al., 2019). In the area of bio‐molecular studies, *L. tarentolae* was firstly exploited to investigate gene amplification (Ouellette et al., 1991; White et al., 1988) and RNA editing in the mitochondrion (Blum et al., 1990). In parallel, *L. tarentolae* was developed as a platform for recombinant protein production (Cantacessi et al., 2015), and then commercialized by Jena Bioscience (Jena, Germany) under the name LEXSY. The LEXSY system allows the expression of target proteins either in a constitutive or inducible form, as intracellular or secretory molecules (https://www.jenabioscience.com/). The strain P10, on which the LEXSY system is based, was likely derived from the TARII/UC strain of the parasite, isolated by Parrot from an Algerian gecko (*T. mauritanica*) (Klatt et al., 2019; Parrot, 1949). Among the variety of microbial and cellular platforms to produce recombinant proteins (e.g., prokaryotes, yeasts, mammalian cells, insect cells), *L. tarentolae* found its niche thanks to some specific characteristics. First, the maintenance and growth of *L. tarentolae* is accomplished at a low cost: promastigotes are easily cultured in aerobic conditions as continuous suspension culture at 26°C, in different synthetic media (Cantacessi et al., 2015; Kushnir et al., 2005). Second, growth characteristics are suitable to scale the production to industrial levels, by growing parasites in bioreactors, with the potential of harvesting high yields of recombinant proteins from engineered strains (Niimi, 2012). Third, *L. tarentolae* presents a protein glycosylation pattern that is very likely to overlap that of pathogenic Trypanosomatidae (Murphy et al., 2020), but is also similar to that of mammals (Cantacessi et al., 2015). Based on the above characteristics, *L. tarentolae* is an interesting system for protein studies (e.g., X‐ray Crystallography) and for the production of protein antigens, for example for sero‐diagnostic applications and vaccine development. To date, the use of this protist to produce antigens for diagnostic application has been limited to experimental studies on antigens from pathogenic species of *Leishmania* or *Trypanosoma* (Rezaei et al., 2019; Rooney et al., 2015) and from viruses (Baechlein et al., 2013; Varotto‐Boccazzi et al., 2021). In this context, a recent paper showed that a recombinant protein produced in *L. tarentolae* allows reliable serological diagnosis of SARS‐CoV‐2 infection (Varotto‐Boccazzi et al., 2021). However, while in the presence of biantennary glycosylation structures, N‐glycosylation in *L. tarentolae* is not completely overlapping that of mammals (Cantacessi et al., 2015). Therefore, the capability of a given viral antigen produced in *L. tarentolae* to match the diagnostic patterns should always be carefully compared with the same antigen expressed from mammalian cells.

In view of its safety and easy culturing, *L. tarentolae* has been investigated as a surrogate pathogen in candidate vaccines, aimed at protecting against human pathogenic *Leishmania* species. In a first seminal paper, Breton et al. (2005) showed that *L. tarentolae* promastigotes are engulfed by DCs in vitro, inducing proper maturation of these cells, with expression of major histocompatibility complex class II (MHCII) and costimulatory molecules. More importantly, this study showed that intraperitoneal administration of live *L. tarentolae* in BALB/c mice determined polarization of the immune response toward Th1 pathway, with significant protection against challenge with *L. donovani*. In successive pre‐clinical studies, live *L. tarentolae* promastigotes were assayed as candidate vaccines in association with adjuvants, with cross‐protective immunity against *L. major* (Haghdoust et al., 2022; Keshavarzian et al., 2020). While the above studies had been performed using non‐engineered strains of *L. tarentolae*, thus exploiting some form of cross‐immunity with human pathogenic species, other studies employed genetically modified strains of *L. tarentolae*, engineered for expression of antigens from human pathogenic leishmanias (Salari et al., 2020; Saljoughian et al., 2013) and/or of immune‐modulating molecules (Montakhab‐Yeganeh et al., 2017), such as proteins from the sand fly saliva (Katebi et al., 2015). These studies generally showed that whole live promastigotes from engineered strains of *L. tarentolae* determined protection in animal models against pathogenic species, including *L. infantum* and *L. major*.

In parallel with the above studies on anti‐*Leishmania* vaccines, *L. tarentolae* was investigated for its potential as a platform to generate anti‐viral vaccines. Targeted viruses include human immunodeficiency virus 1 (Breton et al., 2007), human papillomavirus (Bolhassani et al., 2015) and hepatitis C virus (Ansari et al., 2019). The engineered strains of *L. tarentolae* have so far been assayed only in animal models, either as living vehicles for the antigens (Ansari et al., 2019; Breton et al., 2007), or just as biofactories for antigen production (Bolhassani et al., 2015). The first approach is obviously based on the assumption that the targeting of *L. tarentolae* to DCs should facilitate the delivery of viral antigens to secondary lymphoid organs, ensuring their presentation to CD4+T cells (Breton et al., 2005; Breton et al., 2007). In the second approach, the antigen is administered after purification. Overall, studies above led to encouraging results in animal models, in terms of the generation of virus‐neutralizing antibodies.

## 6 CONCLUDING REMARKS

*Leishmania tarentolae* is a promising protist for its biotechnological applications, of which very little is known regarding its biological cycle, transmission pathways and overall biology. However, the interaction that *L. tarentolae* may have, in endemic areas of canine leishmaniasis, with *L. infantum* and its implications on the pathogenicity and epidemiological cycles of canine and human leishmaniasis are subjects that require further research to better understand natural scenarios. This may open new opportunities for the development of vaccines and/or immune‐protection strategies to control leishmaniases. Yet, this knowledge may be translocated to other areas where *Leishmania* and *Sauroleishmania* occur in sympatry.

Furthermore, recent efforts and studies regarding *L. tarentolae* transmission have demonstrated that this species could have a peripylarian type of development and may colonize the stomodeal valve in *Phlebotomus* spp., supporting transmission via pool‐blood feeding, as seen in mammal‐infecting species of *Leishmania*. Additionally, sand fly prediuresis and consequent contaminative transmission, as well as hosts feeding on infected sand flies, could be another mechanism to infect vertebrates. However, the transmission and development in reptilian hosts and *Sergentomyia* sand flies have yet to be unravelled. Finally, although *L. tarentolae* has historically been considered non‐pathogenic and unlikely to infect mammals, some cultured strains have been shown to be transiently infectious to mammals. The fact that reference laboratory strains are probably non‐infectious even for reptiles, spurs the need for new isolates to fully understand the natural development of *L. tarentolae* in reptiles and in mammals. In addition, this species has been studied as a model for anti‐*Leishmania* vaccines and a platform to generate antiviral vaccines with overall encouraging results in animal models, in terms of the generation of virus‐neutralizing antibodies. The overall picture presented in this review is useful in understanding the implications of the interactions of these sister clades *Leishmania*, which may be applied knowledge to improve diagnostic tools, efficient control and treatment of a neglected disease that is a high burden to our society.

## CONFLICT OF INTEREST

The authors have no conflicts of interest to declare.

## ETHICS STATEMENT

The authors confirm that the ethical policies of the journal, as noted on the journal's author guidelines page, have been adhered to. No ethical approval was required as this is a review article with no original research data.

## AUTHOR CONTRIBUTION

All authors contributed equally to the manuscript.

## Data Availability

The data that support the findings of this study are available from the corresponding author upon request.

## 1

Annex 1. Reference list of the distribution of *Leishmania tarentolae*

**Table:**

Continent	Country	Host	Method of identification	Reference
America	Brazil	Human	Molecular	Novo et al. (2015)
Europe	Portugal	Sand fly	Molecular	Maia et al. (2015)
Europe	Spain	Sand fly	Molecular	Bravo‐Barriga et al. (2016)
Europe	France	Gecko	Culture	Rioux et al. (1969)
Europe	Italy	Gecko	Culture	Pozio et al. (1983)
Europe	Italy	Reptiles	Molecular	Mendoza‐Roldan et al. (2021)
Europe	Italy	Dog	Molecular	Mendoza‐Roldan et al. (2022)
Europe	Italy	Human	Molecular	Pombi et al. (2019); Iatta et al. (2021)
Europe	Italy	Sand fly	Molecular	Latrofa et al. (2018)
Africa	Algeria	Gecko	Culture	Telford (2009)
Africa	Tunisia	Gecko	Culture	Telford (2009)
Africa	Egypt	Gecko	Culture	Wenyon (1921)
Africa	Sudan	Gecko	Culture	Telford (2009)
Africa	Togo	Sand fly	Molecular	Ferlet et al. (2021)
Asia	China	Lizard	Molecular	Zhang et al. (2019)
Asia	China	Snake	Molecular	Chen et al. (2019)
Asia	Turkmenistan	Gecko	Culture	Garnham (1971)

Novo, S. P., Leles, D., Bianucci, R., & Araujo, A. (2015). *Leishmania tarentolae* molecular signatures in a 300 hundred‐years‐old human Brazilian mummy. *Parasites & vectors*, *8*, 72. https://doi.org/10.1186/s13071‐015‐0666‐z

Maia, C., Parreira, R., Cristóvão, J. M., Freitas, F. B., Afonso, M. O., & Campino, L. (2015). Molecular detection of *Leishmania* DNA and identification of blood meals in wild caught phlebotomine sand flies (Diptera: Psychodidae) from southern Portugal. *Parasites & vectors*, *8*, 173. https://doi.org/10.1186/s13071‐015‐0787‐4

Bravo‐Barriga, D., Parreira, R., Maia, C., Blanco‐Ciudad, J., Afonso, M. O., Frontera, E., Campino, L., Pérez‐Martín, J. E., Serrano Aguilera, F. J., & Reina, D. (2016). First molecular detection of *Leishmania tarentolae*‐like DNA in *Sergentomyia minuta* in Spain. *Parasitology research*, *115*(3), 1339–1344. https://doi.org/10.1007/s00436‐015‐4887‐z

Rioux, J. A., Knoepfler, L. P., Martini, A., Callot, J., & Kremer, M. (1969). Présence en France de *Leishmania tarentolae* Wenyon, 1921. Parasite du gecko *Tarentola mauritanica* (L. 1758). Annales de Parasitologie Humaine et Comparée, *44*(1), 115–118.

Pozio, E., Gramiccia, M., Gradoni, L., & Maroli, M. (1983). Hemoflagellates in *Cyrtodactylus kotschyi* (Steindachner, 1870) (Reptilia, Gekkonidae) in Italy. Acta Tropica, 40(4), 399–400.

Mendoza‐Roldan, J. A., Latrofa, M. S., Iatta, R., R S Manoj, R., Panarese, R., Annoscia, G., Pombi, M., Zatelli, A., Beugnet, F., & Otranto, D. (2021). Detection of *Leishmania tarentolae* in lizards, sand flies and dogs in southern Italy, where *Leishmania infantum* is endemic: hindrances and opportunities. *Parasites & vectors*, *14*(1), 461. https://doi.org/10.1186/s13071‐021‐04973‐2

Mendoza‐Roldan, J. A., Latrofa, M. S., Tarallo, V. D., Manoj, R. R., Bezerra‐Santos, M. A., Annoscia, G., Iatta, R., & Otranto, D. (2022). *Leishmania* spp. in Squamata reptiles from the Mediterranean basin. Transboundary and emerging diseases, *69*, 2856–2866. https://doi.org/10.1111/tbed14660.14438

Pombi, M., Giacomi, A., Barlozzari, G., Mendoza‐Roldan, J., Macrì, G., Otranto, D., & Gabrielli, S. (2020). Molecular detection of *Leishmania* (*Sauroleishmania*) *tarentolae* in human blood and *Leishmania* (*Leishmania*) *infantum* in *Sergentomyia minuta*: unexpected host‐parasite contacts. *Medical and veterinary entomology*, *34*(4), 470–475. https://doi.org/10.1111/mve.12464

Iatta, R., Mendoza‐Roldan, J. A., Latrofa, M. S., Cascio, A., Brianti, E., Pombi, M., Gabrielli, S., & Otranto, D. (2021). *Leishmania tarentolae* and *Leishmania infantum* in humans, dogs and cats in the Pelagie archipelago, southern Italy. *PLoS neglected tropical diseases*, *15*(9), e0009817. https://doi.org/10.1371/journal.pntd.0009817

Latrofa, M. S., Iatta, R., Dantas‐Torres, F., Annoscia, G., Gabrielli, S., Pombi, M., Gradoni, L., & Otranto, D. (2018). Detection of *Leishmania infantum* DNA in phlebotomine sand flies from an area where canine leishmaniosis is endemic in southern Italy. *Veterinary parasitology*, *253*, 39–42. https://doi.org/10.1016/j.vetpar.2018.02.006

Telford Jr, S.R. 2009. Hemoparasites of the reptilia: color atlas and text. CRC Press, Florida, USA

Wenyon, C. M. (1920). Observations on the intestinal protozoa of three Egyptian lizards, with a note on a cell‐invading fungus. *Parasitology*, *12*(4), 350‐365.

Ferlet, E., Martinet, J. P., Randrianambinintsoa, F. J., Ravel, C., & Depaquit, J. (2021). Detection of *Leishmania tarentolae* DNA in *Sergentomyia antennata* in Togo. *Journal of Vector Borne Diseases*, *58*(2), 175.

Zhang, J. R., Guo, X. G., Chen, H., Liu, J. L., Gong, X., Chen, D. L., & Chen, J. P. (2019). Pathogenic *Leishmania* spp. detected in lizards from Northwest China using molecular methods. *BMC Veterinary Research*, *15*(1), 1–13.

Chen, H., Li, J., Zhang, J., Guo, X., Liu, J., He, J., … Chen, J. (2019). Multi‐locus characterization and phylogenetic inference of *Leishmania* spp. in snakes from Northwest China. Plos one, 14(4), e0210681.

Garnham, P.C.C. (1971). The genus *Leishmania*. Bulletin Organization Monde Santé Bulletin. World Health Organization 44:477–489.
